# Integrating stable isotopes, parasite, and ring‐reencounter data to quantify migratory connectivity—A case study with Barn Swallows breeding in Switzerland, Germany, Sweden, and Finland

**DOI:** 10.1002/ece3.6061

**Published:** 2020-02-06

**Authors:** Jan A. C. von Rönn, Martin U. Grüebler, Thord Fransson, Ulrich Köppen, Fränzi Korner‐Nievergelt

**Affiliations:** ^1^ Swiss Ornithological Institute Sempach Switzerland; ^2^ Swedish Museum of Natural History Stockholm Sweden; ^3^ Hof Gronow 14 Sundhagen Germany; ^4^ Hiddensee Bird Ringing Centre Güstrow Germany

**Keywords:** Bayesian, *Hirundo rustica*, integrated model, migratory connectivity, wintering area

## Abstract

Ecosystems around the world are connected by seasonal migration. The migrant animals themselves are influenced by migratory connectivity through effects on the individual and the population level. Measuring migratory connectivity is notoriously difficult due to the simple requirement of data conveying information about the nonbreeding distribution of many individuals from several breeding populations. Explicit integration of data derived from different methods increases the precision and the reliability of parameter estimates.

We combine ring‐reencounter, stable isotope, and blood parasite data of Barn Swallows *Hirundo rustica* in a single integrated model to estimate migratory connectivity for three large scale breeding populations across a latitudinal gradient from Central Europe to Scandinavia. To this end, we integrated a non‐Markovian multistate mark‐recovery model for the ring‐reencounter data with normal and binomial mixture models for the stable isotope and parasite data.

The integration of different data sources within a mark‐recapture modeling framework enables the most precise quantification of migratory connectivity on the given broad spatial scale. The results show that northern‐breeding populations and Southern Africa as well as southern‐breeding populations and Western–Central Africa are more strongly connected through Barn Swallow migration than central European breeding populations with any of the African wintering areas. The nonbreeding distribution of Barn Swallows from central European breeding populations seems to be a mixture of those populations breeding further north and south, indicating a migratory divide.

## Graphical abstract

For three breeding populations, an integrated analyses of stable isotopes, ring‐reencounter, and parasite data resulted in precise estimates of their spatial distribution during the nonbreeding period. The northern and southern populations are clearly higher connected to one of the nonbreeding areas compared to the population in the center of the study area.

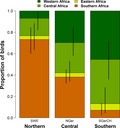

## 1 INTRODUCTION

Migratory connectivity describes the extent to which individuals of the same populations living in close vicinity to each other all year long or whether they are mixing with individuals from other populations at some point of their annual cycle, or in other words it is the degree to which different geographic areas are connected by migrating animals (Webster, Marra, Haig, Bensch, & Holmes, 2002). Given the spatial scale in focus, the spread and the mixing of migrant populations in the wintering area—the degree of connectivity—can vary from *weak* or *diffuse* with high population spread and high degree of mixing to *strong* with low population spread and low mixing in the wintering area (Finch, Butler, Franco, & Cresswell, 2017). Migratory connectivity is a continuous phenomenon expected to vary largely between species, populations, temporal, and spatial scales (Bauer, Lisovski, & Hahn, 2016; Cohen et al., 2018).

Migratory animals face a huge diversity of selection pressures throughout the year. The knowledge of the distribution of populations or groups of individuals of a migratory species across the annual cycle has far‐reaching consequences for our understanding of the evolution and ecology of the species. Strong migratory connectivity has the potential that a population adapts to the environments experienced in each season of the annual cycle. As a consequence, gene flow between populations potentially decreases, which ultimately may result in incipient speciation (Shafer & Wolf, 2013).

Ecological conditions experienced at some point of the annual cycle can carry over into future life (cycle) stages and have pronounced effects on survival prospects and reproductive performance of individuals (van Gils et al., 2016). Variation in the ecological conditions experienced throughout the year by individuals from the same breeding population is expected to be much larger when migratory connectivity within the focal population is weak compared to when it is strong. Therefore, the degree of migratory connectivity is vital for our understanding of population dynamics and eventually the conservation of migratory species (Woodworth, Wheelwright, Newman, Schaub, & Norris, 2017).

Understanding migratory connectivity of populations will ultimately help understanding the spread or the distribution of infectious diseases impacting natural ecosystems, livestock, and human health (Altizer, Ostfeld, Johnson, Kutz, & Harvell, 2013; Bauer & Hoye, 2014).

The study of migratory connectivity at large scales is difficult. Several methods have been used to study the journeys of long‐distance migrants. These methods include extrinsic markers such as individual marking (Bauthian, Gossmann, Ferrand, & Julliard, 2007), VHF (Taylor et al., 2017) and GPS (Klaassen, Strandberg, Hake, & Alterstam, 2008) telemetry and light‐level geolocators (Liechti et al., 2015; Stutchbury et al., 2009). Intrinsic approaches include morphology (Lopes, Marques, & Wennerberg, 2006), genetics (Lovette, Clegg, & Smith, 2004), parasites (Fallon, Fleischer, & Graves, 2006) and variation of trace elements (Szép et al., 2009) and stable isotopes (e.g., Hénaux, Powell, Vrtiska, & Hobson, 2012) in different tissues. Each method has its own advantages and shortcomings. Reencounters of marked individuals are biased because the probability of finding and reporting a marked individual strongly varies geographically (Thorup, Korner‐Nievergelt, Cohen, & Baillie, 2014). The rather large weight of GPS devices prevents them from being used on small animals. Light‐level geolocators require that the individual is recaptured after its migration, which can produce a sampling bias because individuals that die or disperse are not recaptured. Morphology and/or genetic markers are sometimes used to determine the breeding population origin of individuals during the nonbreeding season (Ergen, Chernetsov, Lundberg, Åkesson, & Bensch, 2017; Lopes et al., 2006). However, it is usually not possible to unambiguously assign individuals to a given breeding population due to overlapping phenotypes or low levels of genetic differentiation between populations (von Rönn, Shafer, & Wolf, 2016). Parasites, trace elements, and stable isotopes often contain some geographic information but the spatial resolution is low (Fallon et al., 2006; von Rönn, Harrod, Bensch, & Wolf, 2015; Szép et al., 2009), especially when compared to modern tracking devices.

Commonly only a single data source is used to describe the distribution of individuals of a migratory species at different times of the annual cycle, even though it has long been recognized that the combination of different data sources is important in the study of migratory connectivity (e.g., Boulet & Norris, 2006). A common feature of many studies using two or more data sources is a sequential way of analysis of each data source more or less independently from each other (Procházka, Wilgenburg, Neto, Yosef, & Hobson, 2013; Ryder, Fox, & Marra, 2011; Strandberg, Klaassen, & Thorup, 2009). Only quite recently, it is recognized that a formal integration of different data sets in one model has at least two advantages over the sequential, independent analyses of the single data sets. Firstly, it enables estimating otherwise not identifiable parameters, and secondly, it increases the precision of the parameter estimates (e.g., Schaub & Abadi, 2011).

Hobson, Møller, and Van Wilgenburg (2012), one of the first studies who formally combined different data sets for the estimation of migratory connectivity, used the distribution of ring recoveries as prior for the analyses of stable isotope data. Thereby, they assumed that the spatial distribution of ring recoveries reflects the spatial distribution of the birds. Consequently, the estimates could be biased because the probability that the ring of a dead bird is found and reported to a ringing scheme may be spatially heterogeneous (Korner‐Nievergelt et al., 2010; Thorup et al., 2014). Analyses of ring‐reencounter data using a mark‐recapture and recovery model allow for taking spatial heterogeneity of ring recovery probability into account when inferring spatial distribution of the birds (Bauthian et al., 2007; Korner‐Nievergelt et al., 2010).

In this study, we provide a mark‐recapture modeling framework which explicitly combines several data sources into a single integrated model to estimate migratory connectivity for multiple populations. We apply this model to estimate migratory connectivity between breeding areas and wintering areas of European Barn Swallows breeding in Switzerland, Germany, Sweden, and Finland. More specifically, we integrate ring‐reencounter data, stable isotope values of feathers, and infection data of avian malaria parasites in a single comprehensive model. Moreover, the model combines the just mentioned data collected during the breeding season in Europe with published data on stable isotopes values in feathers sampled in the wintering area (Szép et al., 2009) and data on avian malaria parasites from birds sampled in sub‐Saharan Africa (Beadell et al., 2009; Bensch et al., 2000; Bonneaud et al., 2009; Chasar et al., 2009; Durrant et al., 2007; Hellgren et al., 2013; Loiseau et al., 2012, 2010; Lutz et al., 2015; Marzal et al., 2011; Mendes et al., 2013; Sorensen et al., 2016; Waldenström, Bensch, Kiboi, Hasselquist, & Ottosson, 2002).

## 2 MATERIAL AND METHODS

We collated three types of data potentially harboring information about the geographic distribution of Barn Swallows in Africa: (a) ringing and reencounter data of the Barn Swallows provided by the respective ringing schemes in Finland, Sweden, Germany, and Switzerland (Table 1), (b) stable carbon and nitrogen isotope values in feathers grown in the wintering area (Evans, Waldron, & Bradbury, 2003; Hobson, Møller, et al., 2012; Møller & Hobson, 2004; von Rönn et al., 2015), and (c) infection data of avian malaria parasites of the genus *Plasmodium* known to be transmitted in Africa (Bensch, Hellgren, & Perez‐Tris, 2009; von Rönn et al., 2015).

**Table 1:** Description of ringing and reencounter data of European Barn Swallows from ringing schemes used in this study

Ringing scheme	Switzerland	Radolfzell^a^	Helgoland^a^	Hiddensee^a^	Sweden	Finland
Total years covered	1925–2013	1948–2013	1912–2013	1964–2013	1913–2013	1913–2013
Number of ringed individuals^b^	79,389^c^	14,510^d^	110,488^d^	118,686^e^	89,558^f^	266,839^g^
Total number of reencounters	17	17	36	13	13	53
Number of reencounters with known number of ringed individuals	6	2	8	13	8	53

Note

The numbers of reencounters refer to individuals reencountered in the wintering areas in Africa south of the Sahara.

a All three Ringing Schemes from Germany.

b Years covered with known number of ringed individuals

c All chicks from 1925 to 2012, full grown individuals from 2008 to 2012.

d 2000–2012

e 1964–2012

f 1969–2012

g 1913–2012

Similar to earlier studies, we defined three different geographical breeding areas which correspond to Northern (Sweden, SWE, Finland, FI), Central (Northern Germany (>52°N), NGer), and Southern (Southern Germany (<52°N) and Switzerland, SGerCH) European populations (von Rönn et al., 2015, 2016). The distinction between Central (NGer) and Southern (SGerCH) breeding areas is supported by the similarity between the distribution of feather stable carbon isotope values of Barn Swallows breeding in Thuringia (see stable isotope data) and Saxony (Hobson, Møller, et al., 2012) on the one hand, and the one in Klettgau and Wauwilermoos on the other hand (Evans et al., 2003; von Rönn et al., 2015). Our study areas in Europe cover a latitudinal gradient along which earlier ring‐reencounter studies indicated differences in wintering area distribution (Ambrosini, Møller, & Saino, 2009), they include a migratory divide within a pattern of a leap‐frog migration (Salomonsen, 1955) between populations breeding further north and south (Bønløkke et al., 2006; Fransson & Pettersson, 2001; Liechti et al., 2015; Valkama et al., 2014).

**Figure 1:**
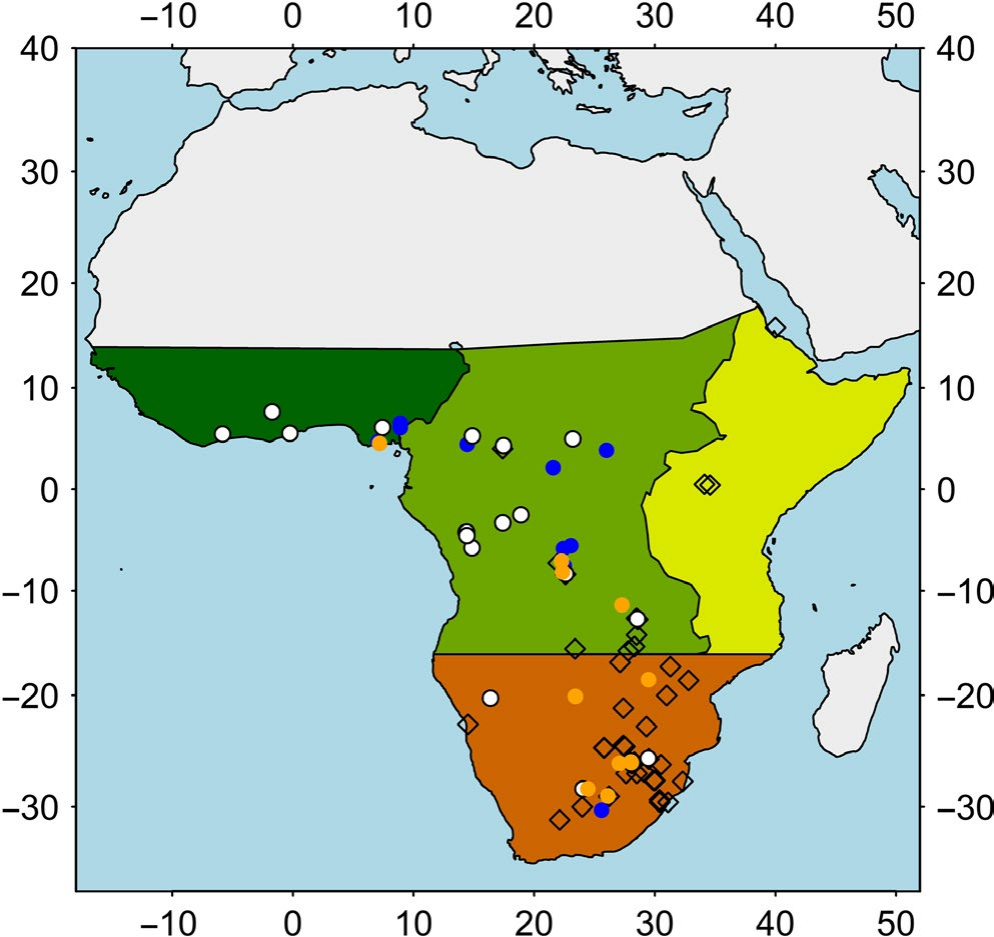
Ring‐reencounters of Barn Swallows in the African wintering area between ringed during the breeding season in Northern (SWE: orange dots; FIN: open diamonds), Central (white dots), and Southern (blue dots) breeding areas. African wintering areas as defined in this study. Dark green—Western Africa, light green—Central Africa, yellow—Eastern Africa, and orange—Southern Africa

We defined four large wintering areas in Africa south of the Sahara (Figure 1), (a) Western Africa from Nigeria to the west, (b) Central Africa consisting of the Congo Basin from the Atlantic Ocean to the Great Rift Valley in the east and north of 15.4°S, (c) Eastern Africa from north of 15.4°S to the Red Sea and the Indian Ocean, east of the Great Rift Valley, and (d) Southern Africa, everything south of 15.4°S.

### 2.1 Ring‐reencounter data

The ringing and reencounter data from Central and Southern European populations (Germany and Switzerland) were split into three groups in the following way (a) spring: all full‐grown birds ringed in March, April, and May, (b) breeding: all birds ringed as chicks and all full‐grown birds ringed during June and July, and (c) autumn: chicks and all full‐grown birds ringed between August and October, respectively. We are aware of the fact that some full grown individuals ringed in May and August might truly belong to the “breeding group.” However, in this way we made sure that no migratory birds were counted to the breeding populations in which we were mainly interested. The ringing and recovery data from Northern populations (Sweden) were not split into additional seasonal groups (spring, breeding, autumn) because all the birds ringed (or recovered) in Sweden are most probably originating from Sweden or Norway or at least are expected to have similar African wintering distributions (Ambrosini et al., 2009; Bakken, Runde, Tjørve, & Koblik, 2006; Fransson, Pettersson, Österblom, & Hall‐Karlsson, 2001–2008).

Additionally, we used ringing and recovery data of Barn Swallows from Finland which we also did not split into seasonal groups (spring, breeding, autumn). The ringing and recovery data from Finland and Sweden were not merged because (a) we only have feather stable isotope and avian malaria infection data from Sweden (von Rönn et al., 2015) and (b) due to some evidence suggesting that breeding birds originating from these two countries slightly differ in their longitudinal distribution in Africa during the nonbreeding season, with individuals from Finland wintering on average further east than Swedish Barn Swallows (Fransson et al., 2001–2008; Valkama et al., 2014).

We grouped the ringing and reencounter data into eight groups of individuals representing different combinations of geographical areas where and seasons when these individuals were marked: Northern (Sweden, SWE, and Finland, FIN, separately), Central‐spring, Central‐breeding, NGer, Central‐autumn, Southern‐spring, Southern‐breeding, SGerCH, and Southern‐autumn. These eight groups are expected to have different spatial distribution in Africa during the nonbreeding period. We use the term *population* for all groups with known breeding origin (Northern, Central‐breeding, and Southern‐breeding population), and the term *groups* for all groups of birds with unknown breeding origin (birds ringed during spring and autumn migration). Here, we were primarily interested in connectivity between breeding and wintering area and not between migratory area and wintering area. However, we included the groups marked during spring and autumn migration in order to increase the precision of the parameter estimates (Korner‐Nievergelt, Schaub, Thorup, Vock, & Kania, 2009).

Because we aimed at estimating migratory connectivity between breeding and wintering areas, we only included birds reencountered in the African wintering area south of the Sahara from November to February, that is, the time when Barn Swallows are largely resident during the nonbreeding season (Liechti et al., 2015).

### 2.2 Stable isotope data

We measured stable carbon (δ^13^C) and nitrogen (δ^15^N) isotope values in feathers grown in the wintering area as described elsewhere (von Rönn et al., 2015). In addition, we also included data (*n* = 25 individuals) from another sampling site in Küllstedt, Thuringia, Germany, 51°16'N 10°17'E (Figure S1) measured in the same way. The samples from the different study sites were merged into the three larger breeding area populations of Barns Swallows described above: (a) Northern (SWE): Kvismaren, (b Central (NGer): Itzehoe and Greifswalder Oie, (c) Southern (SGerCH): Küllstedt, Klettgau, and Wauwiler Moos (Figures S1 and S2).

### 2.3 Parasite data

We collected blood samples from adult breeding birds in Sweden, Germany, and Switzerland (for details see von Rönn et al., 2015) and used a PCR‐based detection method for avian malaria blood parasites described by Hellgren, Waldenstrom, and Bensch (2004). However, in this study we only used data on mitochondrial lineages of the genus *Plasmodium* (GRW02, GRW09, SYBOR21, LINOLI1, PSEGRI1, Table 3) which are most probably transmitted in Africa (Bensch et al., 2009; von Rönn et al., 2015). To collect information about the spatial distribution and prevalence of these *Plasmodium* lineages, we searched the MalAvi‐database (http://mbio-serv2.mbioekol.lu.se/Malavi/index.html) for studies conducted in Africa south of Saharan desert (Beadell et al., 2009; Bensch et al., 2000; Bonneaud et al., 2009; Chasar et al., 2009; Durrant et al., 2007; Hellgren et al., 2013; Loiseau et al., 2012, 2010; Lutz et al., 2015; Marzal et al., 2011; Mendes et al., 2013; Sorensen et al., 2016; Waldenström et al., 2002). From these studies, we took the number of birds sampled (resident African species and Palearctic migrants) and the number of individuals infected with the focal lineages. If this was not directly possible, we asked the corresponding authors of the respective studies to provide such data for our five focal *Plasmodium* lineages (Table 3). We used these data to estimate wintering area‐specific prevalence of each *Plasmodium* lineage, but irrespective of the host species.

### 2.4 MODEL

#### 2.4.1 Integration of the different data sources

The different data sources were combined within an integrated two‐level migratory connectivity model. Separate likelihoods were formulated for each of six data sets (ring‐reencounters with known number of ringed birds (2 age classes), ring‐reencounters with unknown numbers of ringed birds (2 age classes, see below), stable isotope data and parasite data), while all models shared the connectivity parameters *m_i,k_* (Table 2). The connectivity parameters measure the proportion of individuals of each population *i* (pop*_i_*) which migrate to each of the four different wintering areas (*k* = 1:4, the ":" sign means "to"). For a given population (*i*) the *m_i_*_,1:4_ sum to 1, $\sum_{k=1}^{4}m_{i,k}=1$. We assumed that the six data sets were samples of the same super‐population. That is, we assumed that the true proportions *m_i,k_* were independent of whether the number of marked birds was known or not, independent of whether they were marked as juveniles or adults, and independent of whether the individuals were sampled by ring‐reencounters, stable isotopes, or parasites. Note that *m_i,k_*_=1:4_ is a characteristic of the population (*i*) not a characteristic of an individual bird. To construct prior distributions for the migratory connectivity parameters, we used an auxiliary parameter $m_{i,k}^{*}$ for which we specified independent flat beta distributions Beta(1,1). Then, we scaled these parameters so that they summed to one within each population: $m_{i,k}=m_{i,k}^{*}/\text{sum}\left(m_{i,1:4}^{*}\right)$.

**Table 2:** Notation and definition of data, parameters and indices used in the models. We use bold letters for indicating vectors or matrices

Notation	Definition and description
Data	
**R** ^ad,unknown^	Matrix (8 × 4) of the number of ring‐reencounters from each group *i* in four nonbreeding areas. This matrix contains data of birds ringed as adults in those areas and during the periods when the number of marked birds is not known.
**R** ^juv,unknown^	Matrix (8 × 4) of the number of ring‐reencounters from each group *i* in four wintering areas. This matrix contains data of birds ringed as juveniles (pulli) in those areas and during the periods when the number of marked birds is not known.
**R** ^ad,known^	Matrix (8 × 41) of the number of ring‐reencounters from each group *i* in four wintering areas. This matrix contains data of birds ringed as adults in those areas and during the periods when the number of marked birds is known. The last column contains the number of never recovered individuals for each group.
**R** ^juv,known^	Matrix (8 × 41) of the number of ring‐reencounters from each group *i* in four wintering areas. This matrix contains data of birds ringed as juveniles (pulli) in those areas and during the periods when the number of marked birds is known. The last column contains the number of never recovered individuals for each group.
**Y**	a two‐columns matrix containing the two stable isotope measurements δ^13^C and δ^15^N for 859 individuals
**G** ^nb^	Matrix (4 nonbreeding areas × 5 parasite species); number of infected blood samples from the wintering areas
**H** ^nb^	Matrix (4 nonbreeding areas × 5 parasite species); total number of blood samples from the wintering areas
**G** ^b^	Matrix (3 breeding populations × 5 parasite species); number of infected blood samples from the breeding populations
**H** ^b^	Matrix (3 breeding populations × 5 parasite species); total number of blood samples from the breeding populations
Parameters	
*m_i,k_*	Connectivity parameter: proportion of birds from population (or group) *i* that are in area *k* during the months November to February
$r_{k}^{\mathrm{ad}}$	Probability that an individual that has been ringed as adult and that is using wintering area *k* is reencountered at least once during a nonbreeding period.
$r_{k}^{\mathrm{juv}}$	Probability that an individual that has been ringed as a pulli and that is using wintering area *k* is reencountered at least once during a nonbreeding period.
**μ**	2 (wintering areas) x 2 (stable isotope variables) matrix containing the means for each stable isotope variables (δ^13^C and δ^15^N) for two wintering areas. Due to lacking pronounced differences in stable isotope signatures between Western, Central, and Eastern Africa, we pooled these 3 nonbreeding areas and only separated Southern Africa from pooled ones further north.
**Σ**	2 × 2 × 2 array of variances and covariances for the two stable isotope variables (δ^13^C and δ^15^N) in the two wintering areas (Western, Central and Eastern Africa vs. Southern Africa)
$f_{k,p}^{\text{nb}}$	Prevalence of parasite species *p* in wintering area *k*
$f_{\mathrm{ip}}^{b}$	Prevalence of parasite species *p* in breeding population *i*
*a*	Ratio of prevalences between wintering and breeding area.
Indices	
*i*	Group of birds marked during the same season (spring, breeding or autumn) in the same area (see text). Total number of groups is 8. The groups marked during the breeding season are called "populations."
Pop	Indicator of the breeding population (Southern, Central, and Northern) for each of the 859 individuals with stable isotope measurements.
*k*	wintering areas: 1 = Western Africa, 2 = Central Africa, 3 = Eastern Africa, 4 = Southern Africa
*t*	year after marking, 1 = first nonbreeding period after marking (around half a year after marking), 2 = second nonbreeding period after marking, …, 10 = 10th nonbreeding period after marking
*v*	index of the cell in the multinomial model: 4 (nonbreeding areas) x 10 (years) cells
*j*	individual within the stable isotope data, *j* = 1, …, 859
*p*	parasite lineages, *p* = 1, …, 5

#### 2.4.2 Model for ring‐reencounter data sets

For each data set, we used a different adaptation of the mark‐reencounter model presented earlier for the Common Nightingale *Luscinia megarhynchos* (Korner‐Nievergelt, Liechti, & Hahn, 2012). The model is a non‐Markovian formulation of the multistate model described by Arnason (1973) and Schwarz, Schweigert, and Arnason (1993).

The number of reencounters of birds marked in breeding population (*i*) and reencountered at least once in one of the four wintering areas (*k*) during the nonbreeding season plus the number of marked birds (Table S1) which were never reencountered $R_{i,1:5}^{\mathrm{age},\;\text{known}}$ was assumed to be multinomially distributed with cell probabilities depending on the migratory connectivity parameters *m_i,k_* and the area‐specific probability that a marked bird that stays in area *k* during the wintering season is found and reported, $r_{k}^{\text{age}}$. In this way, we accounted for spatially differences in reencounter probabilities as well as for different reencounter probabilities between the age classes (juv: ringed as pulli; ad: ringed as fully grown). Reencounter probability is expected to differ between the age classes because of birds ringed as pulli a smaller proportion will arrive in the winter area (where reencounter takes place) compared to birds ringed as full grown (Grüebler, Korner‐Nievergelt, & Naef‐Daenzer, 2014).

$$R_{i,1:5}^{\text{age, known}}∼\text{Multinom}\left(P_{i,1:5},\;N_{i}\right)\;\text{with}\;P_{i,k}=m_{i,k}r_{k}^{\text{age}}\;\text{and}\;P_{i,5}=1-\sum_{k=1}^{4}P_{i,k}.$$

The cell probabilities *P_i,k_* were equal to the product of the proportion of birds from population *i* that migrated to area *k*, *m_i,k_*, and the probability that a marked bird that migrates to area *k* is reencountered in this area at least once during its lifetime, *P_i,k_* = *r_k_m_i,k_*. The last cell probability was one minus the sum of the first four, that is, the probability that a marked bird is never reencountered again.

The reencounter numbers of birds marked in population *i* with unknown numbers of marked birds (Table S2), $R_{i,1:4}^{\text{age, unknown}}$ was also assumed to be multinomially distributed. However, the last cell probability was missing and the first four cell probabilities were scaled so that they summed to one.

The sum of the ring‐reencounters was used as the size parameter:

$$R_{i,1:4}^{\text{age, unknown}}∼\text{Multinom}\;\left(Q_{i,1:4},\sum_{v=1}^{4}R_{i,v}^{\text{age},\;\text{unknown}}\right).$$

The first four cell probabilities were scaled so that they summed to one: $Q_{i,k}=P_{i,k}/\sum_{v=1}^{4}P_{i,v}$. Note that such a multinomial model for reencounter data with an unknown number of marked birds is not identifiable if no other information on recovery probability is available. However, when integrating this model into the above model, the data with unknown number of marked individuals can provide information about connectivity parameters *m_i,k_* for more populations than data with known number of marked individuals exists.

For the reencounter probabilities, $r_{k}^{\text{age}}$, that is, the probabilities that a marked individual is found in area *k* and reported to the ringing scheme, we assumed independent uniform prior distributions, $r_{k}^{\text{age}}$ ~ Unif(0,1). Because the number of reencounters in Eastern Africa was low, we assumed the reencounter probability in Eastern Africa to be equal to the one in Central Africa, *r*_Eastern Africa_ = *r*_Central Africa_.

#### 2.4.3 Model for stable isotope data

The δ^13^C and δ^15^N values of each individual *j*, **y** *_j _*= (δ^13^C*_j_*, δ^15^N*_j_*), were modeled (after standardization) as a mixture of bivariate normal distributions. This model naturally accounts for the fact that the single individuals were not assigned with certainty to one of the wintering areas, that is, they belonged to each of the wintering areas with a probability that depended on their isotopic signature. The means and covariances of the bivariate normal distributions depended on the latent indicator variable for the nonbreeding area, *W_j_*, to which individuals were assigned with the probability vector **v**

$$y_{j}∼\;\text{MVNorm}\left(\mu \left(W_{j}\right),\Sigma \left(W_{j}\right)\right)$$

$$W_{j}∼\text{Categorical}\left(v_{\text{pop}[j]}\right)$$

Given the spatial scale of the different wintering areas as they are defined in this study and the presumed variation of carbon and nitrogen stable isotope values in Africa (Hobson, Van Wilgenburg, et al., 2012), we only use the stable isotope data to discriminate between two larger areas within Africa, which is Southern Africa versus the three wintering areas further north (Evans et al., 2003). Therefore, the probability that individual *j* from breeding population pop*_j_* spent the wintering season in one of the two areas were

$$v_{\text{pop}[j]}=\left(m_{\text{pop}[i]1}+m_{\text{pop}[i]2}+m_{\text{pop}[i]3},\;m_{\text{pop}[i]4}\right).$$

We used slightly informative prior distributions for the means of the stable isotope measurements for each nonbreeding area, **μ**(*W*). These prior distributions were obtained as follows: The data presented in Szép et al. (2009) were transformed in the same way as ours (subtraction of the mean of our data and division by the standard deviation of our data). Then, we used means and covariances of these feather δ^13^C and δ^15^N values from Southern Africa to construct informative prior distributions for the mean δ^13^C ratios in Southern Africa: *μ*_Southern Africa, δ13C_ ~ Norm(0.97, 0.07), with 0.07 being the standard error of the mean. The mean δ^13^C ratio for the other wintering areas (Western, Central, and Eastern Africa) is expected to be lower than in Southern Africa (Evans et al., 2003; Hobson, Van Wilgenburg, et al., 2012; Still & Powell, 2010), but no real measurements of stable isotopes Barn Swallow feathers from these areas were available. Therefore, we only constrained the estimate of this mean to be lower than the one in Southern Africa, *μ*_WCE Africa, δ13C_ = *μ*_Southern Africa, δ13C_ + *θ, *with *θ* being a negative half‐standard normal distribution *θ* ~ Norm(0,1)[,0].

For the means of δ^15^N, we used standard normal distributions *μ_k,_*_δ15N_ ~ Norm(0,1). These distributions can be considered to be almost noninformative given the z‐transformation of the data.

Additionally, we used the same data from Szép et al. (2009) to construct prior distributions for the covariance between δ^13^C and δ^15^N ratios. Because we expected δ^13^C and δ^15^N ratios to be positively correlated in Southern Africa (von Rönn et al., 2015; Szép et al., 2009), whereas in Western, Central, and Eastern Africa a negative correlation was expected (Bensch, Bengtsson, & Akesson, 2006; von Rönn et al., 2015; Veen et al., 2007), we used slightly informative priors for constructing the covariance matrix for each wintering area, $\sum_{k}=\left[\begin{array}{cc} \sigma_{N}^{2} & \rho_{k}\sigma_{N}\sigma_{C}\\ \rho_{k}\sigma_{N}\sigma_{C} & \sigma_{C}^{2} \end{array}\right]$. The correlation coefficient between δ^13^C and δ^15^N stable values in Szép et al. (2009) was 0.373. Therefore, we used $\rho_{\text{Southern Africa}}=2\left(\rho_{\text{Southern Africa}}^{0}-0.5\right)$ and $\rho_{\text{Southern Africa}}^{0}∼\text{beta}\left(10,4\right)$. For Western, Central, and Eastern Africa, we expected a negative correlation (Bensch et al., 2006; von Rönn et al., 2015; Veen et al., 2007); however, we had no data from Africa available to measure its strength. Therefore, we used a broad distribution with a mean of −0.33, that is, $\rho_{\text{WCE Africa}}=2\left(\rho_{\text{WCE}}^{0}-0.5\right)$ and $\rho_{\text{WCE Africa}}^{0}∼\text{beta}\left(2,4\right)$.

#### 2.4.4 Model for parasite data

Two different data sets of parasite infections were available. The first contained detection—nondetection data of five different lineages of avian malaria parasites in blood samples of various bird species from the different wintering areas. The second data set contained detection—nondetection data of the same five parasite lineages in blood samples from Barn Swallows in the Northern (SWE), Central (NGer), and Southern (SGerCH) breeding populations (Table 3). There is good evidence that Barn Swallows only get infected with these lineages during the time spent in Africa south of the Sahara (Bensch et al., 2009; von Rönn et al., 2015); therefore, we assume that the relative distribution of the parasite lineages in Barn Swallow is proportional to the parasite distribution in their wintering area, for those birds that migrated to the same area.

**Table 3:** Summary of the parasite infection data used in this study

Sampling area	Number of samples	*Plasmodium* lineage				
		SYBOR21	GRW02	GRW09	LINOLI1	PSEGRI1
Breeding area						
Northern (SWE)	50	0	0	1	1	1
Central (NGer)	435	12	16	18	0	0
Southern (SGerCH)	90	1	5	8	0	0
Wintering area						
Western Africa	803	1	6	1	0	0
Central Africa	2,767	0	1	74	2	22
Eastern Africa	506	0	0	26	1	0
Southern Africa	660	0	2	0	34	0

Note

In the breeding area, only adult Barn Swallows were sampled (see von Rönn et al., 2015), whereas samples from the wintering area were collected from a number of different bird species (Beadell et al., 2009; Bensch et al., 2000; Bonneaud et al., 2009; Chasar et al., 2009; Durrant et al., 2007; Hellgren et al., 2013; Loiseau et al., 2012, 2010; Lutz et al., 2015; Marzal et al., 2011; Mendes et al., 2013; Sorensen et al., 2016; Waldenström et al., 2002).

First, we estimated the prevalence of each parasite lineage within each wintering area using Binomial models:

$$G_{k,p}^{\text{nb}}∼\text{Binomial}\left(f_{k,p}^{\text{nb}},\;H_{k,p}^{\text{nb}}\right).$$

A similar model was used for the parasite data from the breeding populations:

$$G_{i,p}^{b}∼\text{Binomial}\left(f_{i,p}^{b},\;H_{i,p}^{b}\right).$$

Subsequently, we assumed the following relationship between the prevalence in the wintering areas and the prevalence in the breeding areas:

$$f_{i,p}^{b}=a\sum_{k=1}^{4}\left(f_{k,p}^{\text{nb}}m_{i,k}\right).$$

For the prevalence parameters *f*, we used uniform(0,1) prior distributions and for *a* the positive half‐normal distribution Normal(0, 5)[0,].

Similar to the model for the stable isotope data, but in contrast to the reencounter model, the model for parasite data does not assign a wintering area to each of the individuals with certainty. It is using the individual parasite signatures in the breeding populations to find compatible connectivity parameters, but single individuals are not assigned to one of the wintering areas.

#### 2.4.5 Data sources and information content

First, to assess how well the model parameters were estimated, we compared their posterior distributions with their prior distributions. Posterior distributions similar to the prior distributions indicate weak identifiability of the parameters, that is, lacking or weak information about the specific parameters in the data. In such cases, the prior has a strong influence on the posterior distribution. We used the overlap between the prior and the posterior distribution to quantify the prior influence for each parameter (Garrett & Zeger, 2000; Gimenez, Morgan, & Brooks, 2009).

Second, we quantified the relative contribution of each data type to the final results. To do so, we fitted all sub‐models (ring‐recovery model, stable isotope model, and parasite model) to each data set (ring‐reencounters, stable isotope data, and parasite data) separately. Then, we fitted the complete integrated model including all data sets. The comparison of the posterior distributions of the single data set analyses with the ones from the integrated models shows the relative contribution of each data type to the overall results. We used the overlap of the posterior distribution from the single‐data set model with the one from the integrated model as a measure of the relative contribution of information from the specific data set to the result of the integrated model. This method is proposed and discussed in Korner‐Nievergelt, Prévot, Hahn, Jenni, and Liechti (2017).

#### 2.4.6 Model fitting and assessment of convergence and model fit

The models were fitted in JAGS (http://mcmc-jags.sourceforge.net/) using the package R2jags (Su & Yajima, 2012) and R 3.3.2. Three Markov chains of length 120,000 were simulated. Burn‐in was set to 60,000, and the chains were thinned by six. Convergence was assessed visually and based on the Brooks–Gelman–Rubin statistics (Brooks & Gelman, 1998). All R‐hat values were lower than 1.002. The JAGS code of the complete model is provided in the supplement (Appendix S1).

## 3 RESULTS

Reencounter probabilities were estimated for all four wintering areas, but assumed to be the same in Central and Eastern Africa. As expected, reencounter probabilities in sub‐Saharan Africa were very low (on average 0.0002 for adult and 0.0001 for juvenile Swallows) and similar between different wintering areas (Table S3).

**Figure 2:**
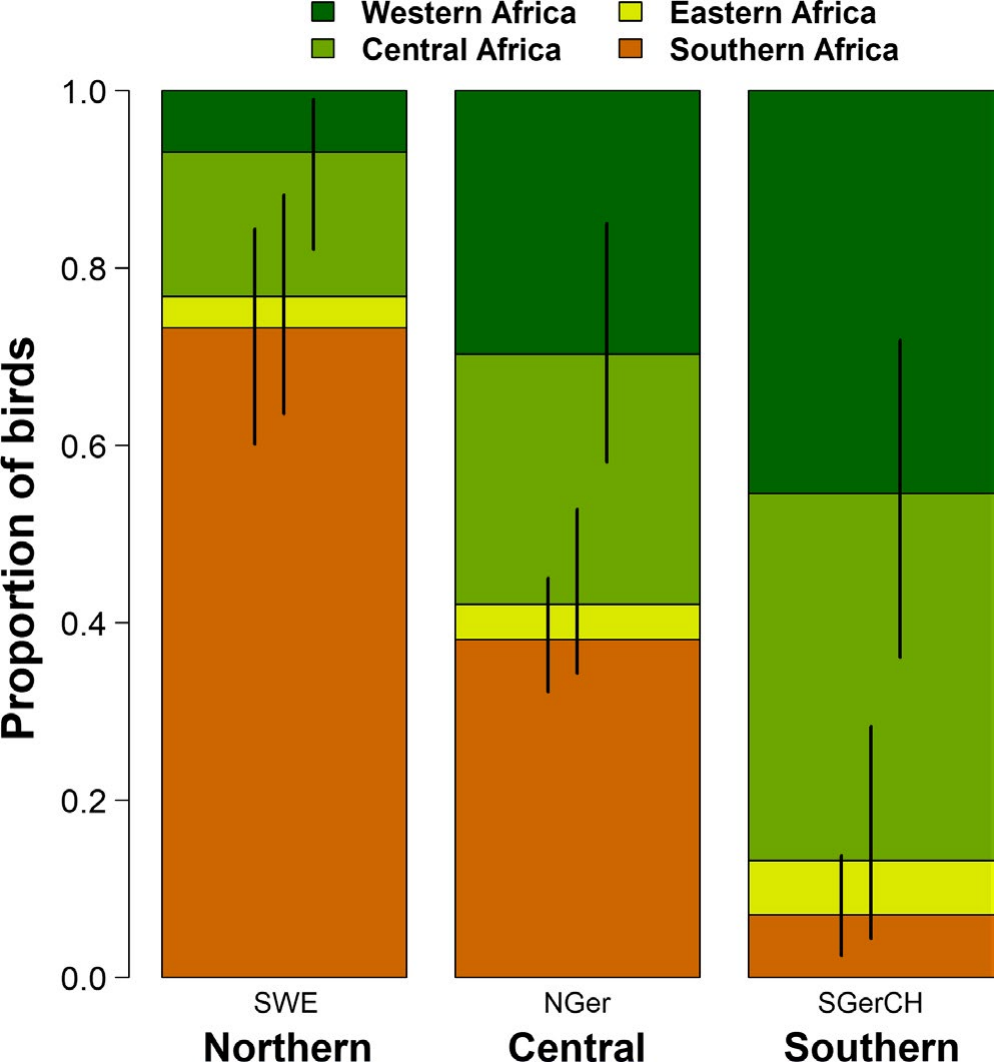
Wintering area distribution and migratory connectivity of European Barn Swallows from Northern (Sweden, SWE), Central (Northern Germany, NGer), and Southern (Germany & Switzerland, SGerCH) breeding populations. Estimated wintering rates (colors) of birds breeding in the three European areas based on the integrated model including ring‐reencounter, stable isotopes, and parasite data. Vertical lines are the corresponding 95% credible intervals

Based on all three available data sources and given the large spatial scale, we estimated African wintering distributions (i.e., the proportion of individuals of each breeding population wintering in a given wintering area) and thereby migratory connectivity for Barn Swallows breeding in three areas across a latitudinal gradient from Switzerland to Fenno‐Scandinavia with high precision (Figure 2, Table S4). We found that most Barn Swallows belonging to Northern populations (Sweden) were wintering in Southern Africa showing relatively high migratory connectivity. A slightly lower degree of migratory connectivity was found for breeding birds from Southern populations (Switzerland and Southern Germany); however, these were predominantly wintering in Western and Central Africa. In contrast, Barn Swallows breeding in the Central study area (Northern Germany) are showing a much lower degree of migratory connectivity by wintering in Western, Central, and Southern Africa in similar proportions. Only a small fraction of breeding birds of these three populations is estimated to spend the winter in Eastern Africa.

Estimated African wintering distributions and migratory connectivity for the additional seasonal groups of birds marked during spring or autumn migration in Central or Southern populations (Germany and Switzerland) and for breeding birds from Finland are shown in Figure S3 and Table S4. Not surprisingly, estimates are less precise for those populations for which (only little) ring‐reencounter data were available. Based predominantly on ring‐reencounter data, breeding birds from Finland behave similarly to the Swedish Barn Swallows by migrating mostly to Southern Africa. The proportion of Finnish birds estimated to spend the wintering period in Eastern Africa tends to be higher than that of Swedish Barn Swallows (Figure S3).

The overlap between the prior and posterior distributions of the recovery probabilities and the connectivity parameters was very low (Tables S3 and S4), indicating that the estimates primarily reflect information in the data (not from the prior).

**Figure 3:**
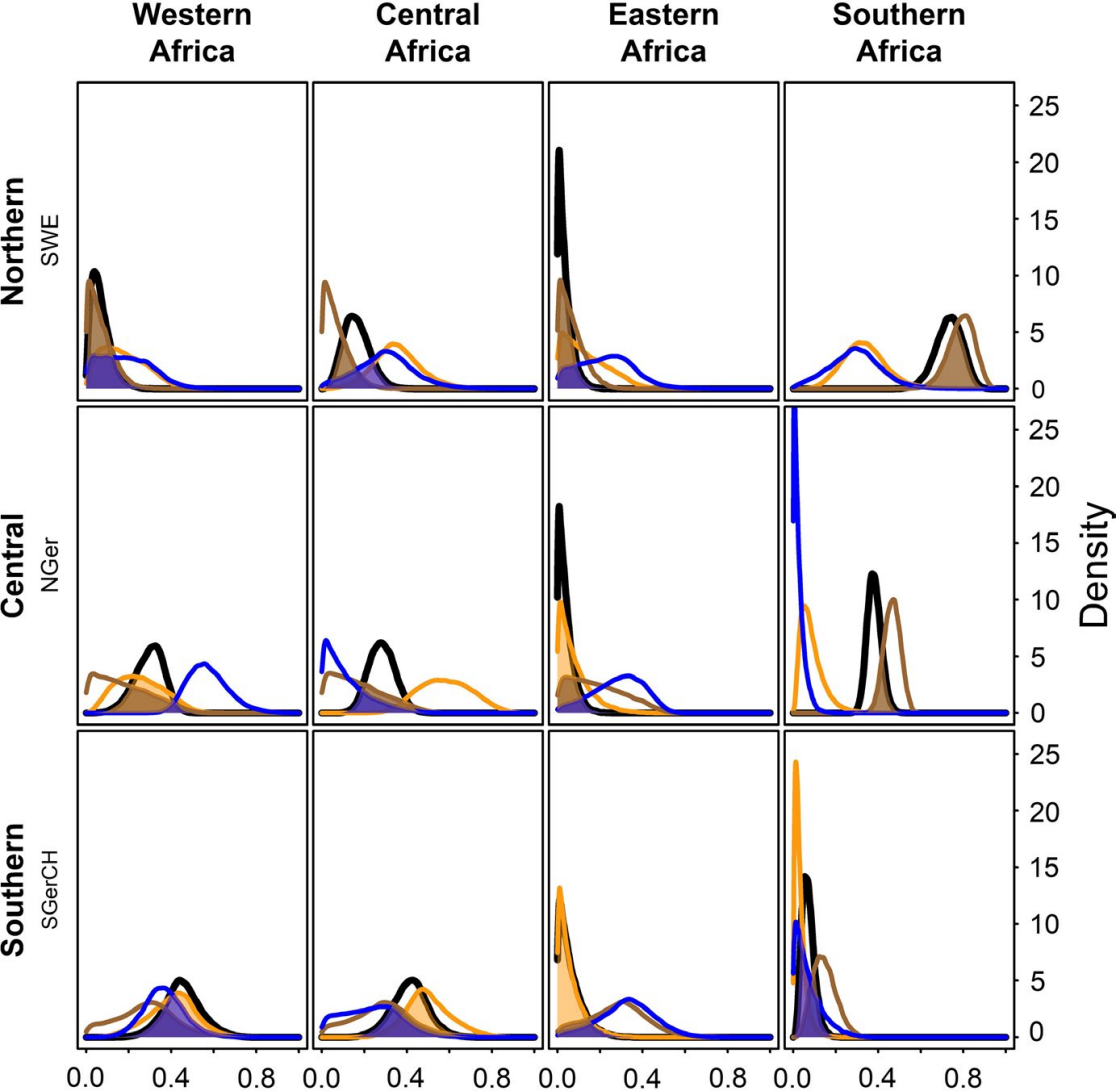
Posterior distributions of the connectivity parameters from the integrated model (including all data sets—black) and the three models fitted to the single data sets (ring‐reencounter—orange, stable isotope—brown, and parasite—blue). The overlap between the single‐data set models and the integrated model is represented by the colored areas. The larger these areas, the stronger the contribution of the specific single data to the combined results

The precision of the estimated connectivity parameter *m_i,k_* was higher in the integrated model compared to the single‐data set models (Figure 3). Further, the estimate of the proportions of migrating individuals to Southern Africa was mostly influenced by the stable isotope data for those two populations of which a high proportion was migrating to Southern Africa (Sweden and Northern Germany, Figure 3). Ring‐reencounters and parasites were more important than the stable isotope data to estimate the proportions of birds in Western, Central, and Eastern Africa. The contribution of ring‐reencounters and parasite data seems to be similar (Figure 3).

## 4 DISCUSSION

The combination of ring‐reencounter data with stable isotope and parasite data resulted in precise estimates of the proportion of birds wintering in different nonbreeding areas for breeding populations of Barn Swallows across a latitudinal gradient from Switzerland to Fenno‐Scandinavia. The results clearly show that the migratory connections from Southern Germany and Switzerland to Western/Central Africa and from Scandinavia to Southern Africa are higher compared to the connections of Northern Germany with any of the African areas.

An earlier study using a yet different method supports our results. In a breeding population in southern Switzerland, Liechti et al. (2015) tracked 92 Barn Swallows with geolocators. They found that 5% of these spent the winter in Southern Africa. This proportion coincides with our estimates of 7% (3%–13%) for our Southern population of Barn Swallows breeding in Southern Germany and Switzerland. Overall, the distributional pattern across the African wintering areas found for the different breeding populations in our study supports earlier views of the distribution of Barn Swallows during residential times of the nonbreeding season (Ambrosini et al., 2009; Hobson, Møller, et al., 2012; von Rönn et al., 2015, 2016; Zwarts, Bijlsma, Kamp, & Wymenga, 2009). The integration of the different data source presented here added direct and precise estimates of migratory connectivity of the focus breeding populations.

Similar to Van Wilgenburg and Hobson (2011) and Rundel et al. (2013), we found that a formal combination of different data types can improve the precision of estimates of migratory connectivity. Two mechanisms are responsible for this improvement. First, the combination of data sets increases sample size because information from more individuals is available. Second, different data types contain qualitatively different information and, therefore, can complement each other as earlier shown for population models (Schaub & Abadi, 2011). In our example, the stable isotope data contained information about the proportion of individuals from different populations that spend the nonbreeding period in similar habitat (Marra, Hobson, & Holmes, 1998). We had independent stable isotope data from Southern Africa informing about the means of and correlation between δ13C and δ15N (Szép et al., 2009). Therefore, the stable isotope data could increase the precision of the estimated proportion of birds migrating to Southern Africa, but they did not contain information on how the rest of the populations were likely distributed among Western, Central, and Eastern Africa. In contrast, ring‐reencounters contributed information for distinguishing between Western, Central, and Eastern Africa because of the precise location information (Baillie, Robinson, Clark, & Redfern, 2009), but they contained less information on the proportion of birds migrating to Southern Africa because of low numbers of ring‐reencounters there from certain breeding populations (Ambrosini et al., 2009). The occurrence of the focal *Plasmodium* lineages differed substantially between Western, Southern, and the two other African wintering areas (see citations in respective Methods section). The same *Plasmodium* lineages predominated in Central and Eastern Africa (Table 3; Durrant et al., 2007). Due to this, we expected parasite data to contribute more information to the estimation of the proportions of birds in Western and Southern Africa compared the two other areas. However, for the estimation of the proportions of birds migrating to Southern Africa the information in the stable isotope data was much stronger than the one in the parasite data (Figure 3), possibly due to low lineage and wintering area‐specific prevalence (Tables S5 and S6).

We used an integrated model fitted in a fully Bayesian framework. The framework was used earlier for the integration of ring‐reencounters and tracking data (Korner‐Nievergelt et al., 2017). Here, we modified this model to integrate ring‐reencounter, stable isotope and parasite data. Our model adds to the current approaches for the estimation of migratory connectivity at least three important aspects: (a) We integrate three different data sources, (b) the uncertainty of the estimates from the single data sets is accounted for in the results of the integrated model, and (c) we account for potential bias due to spatial heterogeneity in reencounter probability (Siriwardena, Wernham, & Baillie, 2004; Thorup et al., 2014).

Based on the combined information from the three data sources, we could quantify that roughly 73% of the Swedish and 77% of the Finnish Barn Swallows spend the winter in Southern Africa (Table S4), breeding birds from Southern populations (SGerCH) winter in high proportions in Western and Central Africa (in total 90%). However, Barn Swallows from the Central populations (NGer) migrate in approximately equal proportions (one third each) to Western, Central, and Southern Africa. Our results showed that Barn Swallows breeding in Northern Germany are more evenly distributed within Africa during the nonbreeding period compared to the populations breeding more to the south and north, respectively. Therefore, migratory connectivity seems to be lower for Barn Swallows from Central populations compared to Southern or Northern populations. Of the latter populations, three quarters of the individuals or more spend the nonbreeding period in Western and Central Africa, and Southern Africa, respectively. As a consequence, the Central population in Northern Germany forms a migratory divide within a pattern of a leap‐frog migration system (Salomonsen, 1955). How broad the migratory divide is, or whether the change in the proportions of birds migrating to the different areas within Africa changes gradually or abruptly along the south–north axis remain open questions. However, the integrated connectivity model presented here could prove useful for answering these questions once spatially continuous data is available along the latitudinal axis.

## CONFLICT OF INTEREST

We have no competing interests to declare.

## AUTHOR CONTRIBUTIONS

JvR and FKN designed the research and analyzed the data. FKN developed the model. TF and UK provided parts of the data. MG, TF, and UK participated in interpretation and discussion of the results. JvR and FKN wrote the manuscript with contribution of MG. Comments and edits from all authors lead to the final draft of the manuscript. All authors approved to publish the manuscript.

## Data Availability

Data and code were uploaded to http://datadryad.org (https://doi.org/10.5061/dryad.3xsj3txbt).
