# Supplementary material for: Integrating stable isotopes, parasite, and ring‐reencounter data to quantify migratory connectivity—A case study with Barn Swallows breeding in Switzerland, Germany, Sweden, and Finland
Source: Ecol Evol. 2020 Feb 6;10(4):2225–37. doi: 10.1002/ece3.6061 (PMC7042758; doi:10.1002/ece3.6061)
Supplement: Supplementary file 1 [file ECE3-10-2225-s001.docx]

**Supporting Information 1**

Integrating stable isotopes, parasite and ring-reencounter data to quantify migratory connectivity - a case study with Barn Swallows breeding in Switzerland, Germany, Sweden and Finland

**Authors**

Jan A. C. von Rönn^1^, Martin U. Grüebler^1^, Thord Fransson^2^, Ulrich Köppen^3^, Fränzi Korner-Nievergelt^1*^

Affiliation

^1^Swiss Ornithological Institute, Seerose 1, CH-6204 Sempach, Switzerland

^2^Swedish Museum of Natural History, Frescativagen 40, 10405 Stockholm, Sweden

^3^Hiddensee Bird Ringing Scheme, An der Mühle 4, 17493 Greifswald, Germany

*corresponding author:

Fränzi Korner-Nievergelt, fraenzi.korner@vogelwarte.ch

**Keywords**

Migratory connectivity, wintering area, integrated model, Bayesian, *Hirundo rustica*

Table S1: Number of reencounters of European Barn Swallows in the wintering areas from different breeding areas in Europe – with known number of ringed individuals (see Material & Methods, Tab. 1). *Southern* corresponds to Southern Germany and Switzerland, *Central* to Northern Germany and *Northern* to Sweden and Finland.

| **Wintering area** | **Breeding area** | | | |
| --- | --- | --- | --- | --- |
|  | **Southern** | **Central** | **Northern (SWE)** | **Northern (FI)** |
| **Western Africa** | 10 | 1 | 0 | 0 |
| **Central Africa** | 3 | 8 | 2 | 6 |
| **Eastern Africa** | 0 | 0 | 0 | 3 |
| **Southern Africa** | 1 | 6 | 6 | 44 |

Table S2: Number of reencounters of European Barn Swallows in the wintering areas from different breeding areas in Europe – with unknown number of ringed individuals (see Material & Methods, Tab. 1). *Southern* corresponds to Southern Germany and Switzerland, *Central* to Northern Germany and *Northern* to Sweden and Finland.

| **Wintering area** | **Breeding area** | | | |
| --- | --- | --- | --- | --- |
|  | **Southern** | **Central** | **Northern (SWE)** | **Northern (FI)** |
| **Western Africa** | 18 | 4 | 1 | - |
| **Central Africa** | 17 | 10 | 1 | - |
| **Eastern Africa** | 1 | 0 | 0 | - |
| **Southern Africa** | 3 | 1 | 3 | - |

Table S3: Reencounter probabilities for juvenile and adult European Barn Swallows in the African wintering areas. Given are posterior means, 95% credible intervals and the overlap between the prior and the posterior distribution for each parameter. Estimates are based on the integrated model including mark-reencounter, isotope and parasite data. Reencounter probabilities in Central and Eastern Africa were assumed to be the same.

| **Wintering area** | **age class** | **mean** | **lower** | **upper** | **overlap** |
| --- | --- | --- | --- | --- | --- |
| **Western Africa** | ad | 0.0002 | 0.00008 | 0.00037 | 0.00084 |
| **Central & Eastern Africa** | ad | 0.0002 | 0.00011 | 0.00033 | 0.00084 |
| **Southern Africa** | ad | 0.0002 | 0.00013 | 0.00025 | 0.00084 |
| **Western Africa** | juv | 0.0001 | 0.00004 | 0.00015 | 0.00084 |
| **Central & Eastern Africa** | juv | 0.0001 | 0.00004 | 0.00014 | 0.00084 |
| **Southern Africa** | juv | 0.0001 | 0.00006 | 0.00015 | 0.00084 |


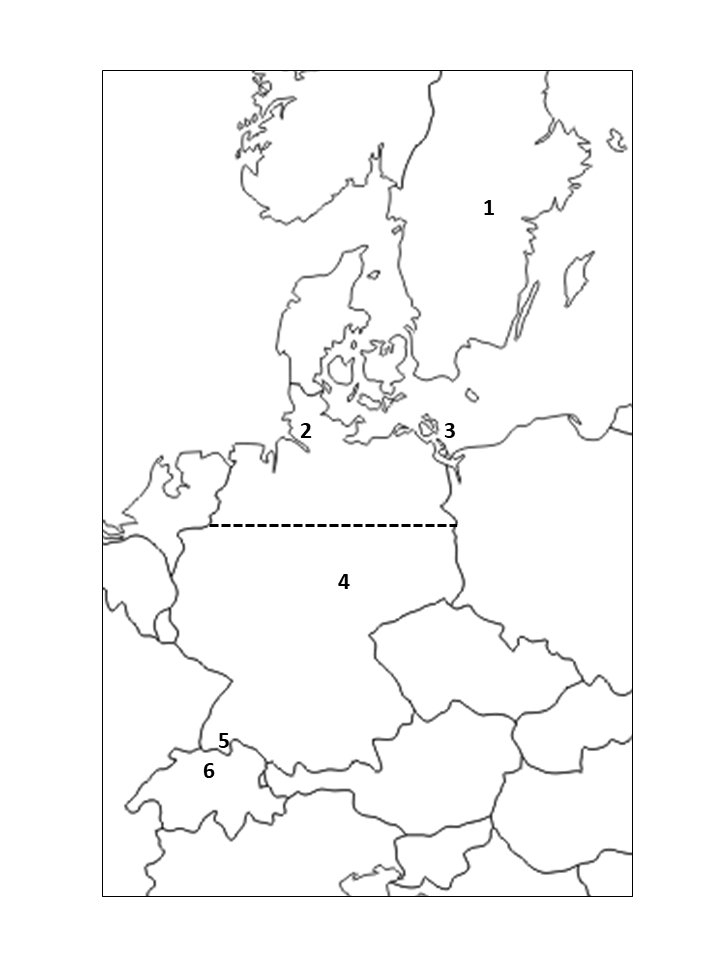


Figure S1: Geographic locations of the sampled Barn Swallow breeding populations (stable isotopes and blood parasites): (1) Kvismaren, Sweden, 59°10’N 15°22’E, (2) Itzehoe, Germany, 53°55’N 09°31’E, (3) Island Greifswalder Oie, Germany, 54°14’N 13°55’E, (4) Küllstedt, Germany, 51°16'N 10°17'E, (5) Klettgau, Germany, 47°39’N 08°25’E, (6) Wauwilermoos, Switzerland, 47°10’N 08°01’E. Blood samples were only collected in Kvismaren, Itzehoe, Island Greifswalder Oie, Klettgau and Wauwilermoos (see von Rönn, Harrod, Bensch & Wolf 2015). The dashed line indicates 52°N.


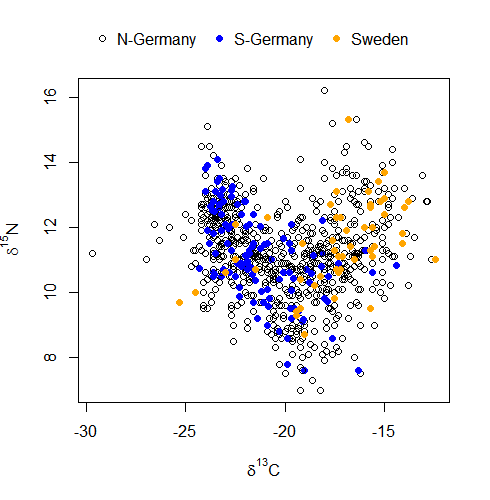


Figure S2: Distribution of nitrogen (δ15N) and carbon (δ13C) stable isotope ratios from feathers grown in the African wintering areas. Orange dots: *Northern* (Sweden, n=50); Open circles: *Central* (Northern Germany, n=695); blue dots: *Southern* (Southern Germany & Switzerland, n=114). Same data as in von Rönn, Harrod, Bensch & Wolf (2015) with data from Küllstedt added (see Fig. S1).


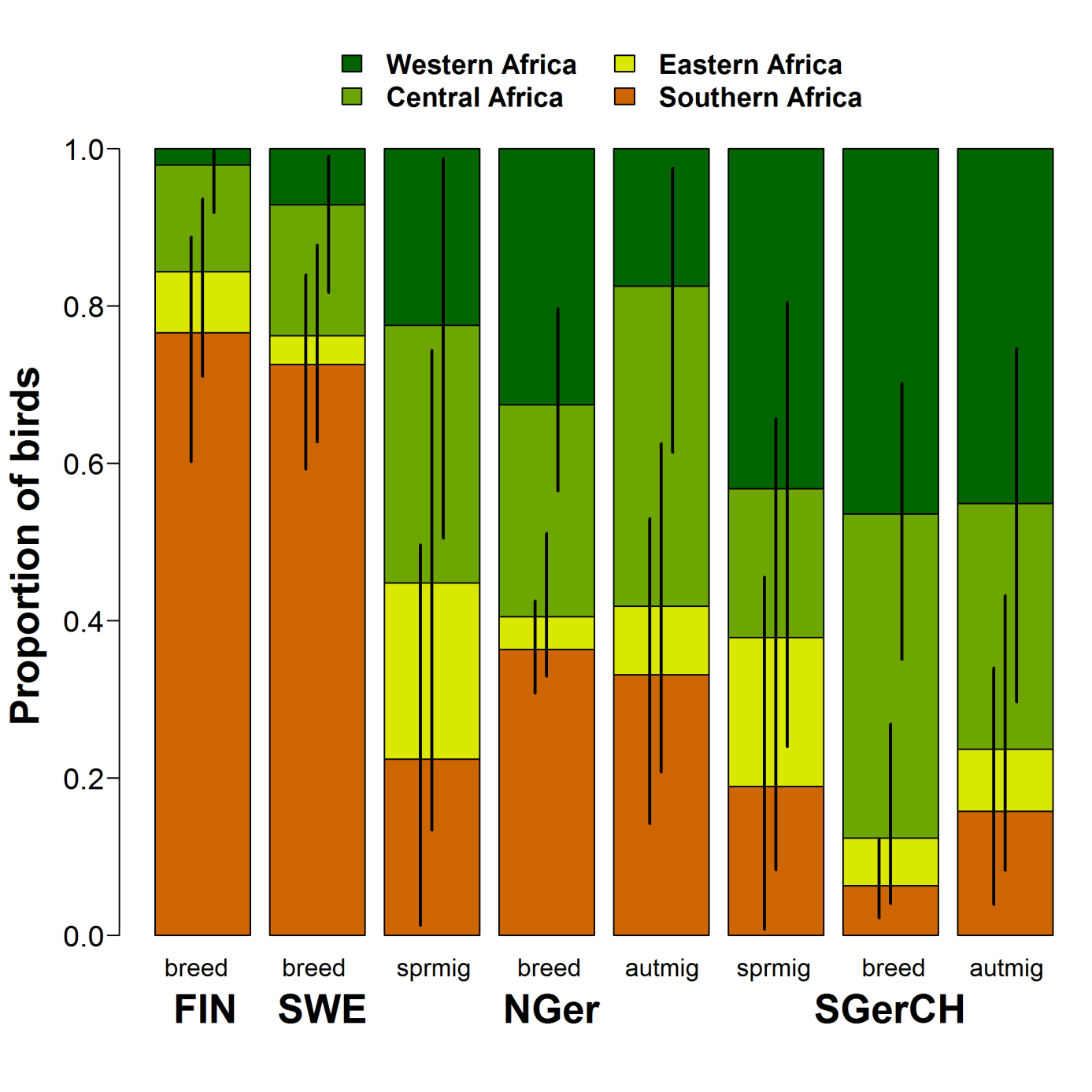


Figure S3: Wintering area distribution and migratory connectivity of European Barn Swallows marked in Finland, Sweden, Northern Germany and Southern Germany & Switzerland. Given are the mean estimated proportions of birds in the respective wintering area (colors) based on the integrated model including ring-reencounter, stable isotope and parasite data. Vertical lines are the corresponding 95% credible intervals. Additional groups compared to Fig. 4: FIN breed – breeding birds from Finland, NGer sprmig – individuals marked during spring migration in Northern Germany, NGer autmig – individuals marked during autumn migration in Northern Germany, SGerCH sprmig – individuals marked during spring migration in Southern Germany and Switzerland, SGerCH autmig – individuals marked during autumn migration in Southern Germany and Switzerland. Same as in Fig. 4: *Northern* = SWE breed, *Central* = NGer breed, *Southern* = SGerCH breed.

Appendix S1: jags code of the model including ring-reencounter, stable isotope and parasite data.

model{

# model for recoveries with known number of ringed

for (i in 1:npop){

for(k in 1:ndest){

pad[i, k] <- m[i,k]*rad[k]

pjuv[i,k] <- m[i,k]*rjuv[k]

}

pad[i, (ndest+1)] <- 1-sum(pad[i,1:ndest])

pjuv[i, (ndest+1)] <- 1-sum(pjuv[i, 1:ndest])

}

# likelihood

for(i in 1:npop){

recmatad[i,1:(ndest+1)]~dmulti(pad[i,1:(ndest+1)], nringedad[i])

recmatjuv[i,1:(ndest+1)]~dmulti(pjuv[i,1:(ndest+1)], nringedjuv[i])

}

# model for recoveries with unknown ringed

for(i in 1:npop){

for(k in 1:ndest){

Epad[i,k] <- m[i,k]*rad[k]/(m[i,1]*rad[1]+m[i,2]*rad[2]+m[i,3]*rad[3]+m[i,4]*rad[4])

Epjuv[i,k] <- m[i,k]*rjuv[k]/(m[i,1]*rjuv[1]+m[i,2]*rjuv[2]+m[i,3]*rjuv[3]+m[i,4]*rjuv[4])

}

recmatruad[i,1:ndest] ~ dmulti(Epad[i,1:ndest], Rad[i])

recmatrujuv[i,1:ndest] ~ dmulti(Epjuv[i,1:ndest], Rjuv[i])

}

# priors

rad[1] ~ dunif(0, 1) # W-Africa

rad[2] ~ dunif(0, 1) # C-Africa

rad[3] <- rad[2] # E-Africa

rad[4] ~ dunif(0, 1) # S-Africa

rjuv[1] ~ dunif(0, 1) # W-Africa

rjuv[2] ~ dunif(0, 1) # C-Africa

rjuv[3] <- rjuv[2] # E-Africa

rjuv[4] ~ dunif(0, 1) # S-Africa

# Parasite model

# within Africa

for(j in 1:nparasites){

for(k in 1 : ndest) {

infw[k,j]~dbin(pwi[k,j], nwinter[k])

}

}

# breeding population

for(i in 1:3){

for(j in 1:nparasites){

infsommer[i,j]~dbin(pinf[i,j], nsommer[i])

}

}

for(i in 1:3){

for(j in 1:nparasites){

for(k in 1:ndest){

pwim[i,j,k] <- pwi[k,j]*a*m[poppar[i],k]

}

pinf[i,j] <- sum(pwim[i,j,1:ndest])

}

}

# initials

a ~ dnorm(0,0.04)I(0,)

for(k in 1:ndest){

for(j in 1:nparasites){

pwi[k,j] ~ dunif(0,1)

}

}

# model for isotopes

for(i in 1 : N) {

y[i,1:2] ~ dmnorm(mu[WiDest[i],1:2], Sigma.inv[WiDest[i],,])

WiDest[i] ~ dcat(miso[pop[i],1:ndestiso]) # miso: proportion of birds from i in k

}

for(i in 1:npop){

miso[i,1] <- sum(m[i,1:3])

miso[i,2] <- m[i,4]

}

for(k in 1:ndestiso){

mu[k,2] ~ dnorm(0, 1) # mean nitrogen stable isotope values for each area k

}

# mean carbon stable isotope values for S-Africa and WCE-Africa

mu[2,1] ~ dnorm(0.97, 200) # for S-Africa

theta1 ~ dnorm(0, 1)I(,0)

mu[1,1] <- mu[2,1] + theta1 # for WCE-Africa

for(k in 1:ndestiso){

Sigma.inv[k,1:2, 1:2] <- inverse(Sigma[k, 1:2, 1:2])

sigma.c[k] ~ dt(0,1,2)I(0,) # sd in d13c

sigma.n[k] ~ dt(0,1,2)I(0,) # sd in d15n

Sigma[k,1,1] <- pow(sigma.c[k],2)

Sigma[k,2,2] <- pow(sigma.n[k],2)

Sigma[k,1,2] <- rho[k]*sigma.c[k]*sigma.n[k]

Sigma[k,2,1] <- Sigma[k,1,2]

}

rho0[1] ~ dbeta(2,4)

rho[1] <- 2*(rho0[1]-0.5)

rho0[2] ~ dbeta(10,4) # for S-Africa

rho[2] <- 2*(rho0[2]-0.5)

for(i in 1:npop){

for(k in 1:ndest){

m0[i,k]~dbeta(1,1)

m[i,k] <- m0[i,k]/sum(m0[i,1:ndest])

} #k

}#i

}
