# Supplementary material for: Integrating stable isotopes, parasite, and ring‐reencounter data to quantify migratory connectivity—A case study with Barn Swallows breeding in Switzerland, Germany, Sweden, and Finland
Source: Ecol Evol. 2020 Feb 6;10(4):2225–37. doi: 10.1002/ece3.6061 (PMC7042758; doi:10.1002/ece3.6061)
Supplement: Supplementary file 2 [file ECE3-10-2225-s002.docx]

**Supporting Information 2**

Integrating stable isotopes, parasite and ring-reencounter data to quantify migratory connectivity - a case study with Barn Swallows breeding in Switzerland, Germany, Sweden and Finland

**Authors**

Jan A. C. von Rönn^1^, Martin U. Grüebler^1^, Thord Fransson^2^, Ulrich Köppen^3^, Fränzi Korner-Nievergelt^1*^

Affiliation

^1^Swiss Ornithological Institute, Seerose 1, CH-6204 Sempach, Switzerland

^2^Swedish Museum of Natural History, Frescativagen 40, 10405 Stockholm, Sweden

^3^Hiddensee Bird Ringing Scheme, An der Mühle 4, 17493 Greifswald, Germany

*corresponding author:

Fränzi Korner-Nievergelt, fraenzi.korner@vogelwarte.ch

Keywords

Migratory connectivity, wintering area, integrated model, Bayesian, Hirundo rustica

Appendix Table 4: Wintering area distribution and migratory connectivity of European Barn Swallows marked in Finland, Sweden, Northern Germany and Southern Germany & Switzerland. Given are the estimated proportions (posterior mean, 95% credible interval and the overlap between the prior and the posterior distribution for each parameter) of birds in the respective non-breeding area based on the model including ring-reencounter, stable isotope and parasite data. Estimates are given for the focal Northern, Central and Southern breeding populations (SWE, NGer-breeding, SGerCH-breeding), for Barn Swallows from Finland and for the individuals marked during spring and autumn migration seasons in the Central and Southern part of the study area (NGer-spring/-autumn, Southern-spring/-autumn).

|  | **Wintering area** | | **Western Africa** | | | | **Central Africa** | | | | **Eastern Africa** | | | | **Southern Africa** | | | |
| --- | --- | --- | --- | --- | --- | --- | --- | --- | --- | --- | --- | --- | --- | --- | --- | --- | --- | --- |
| **marking area** | **season** | **data sources** | **mean** | **lower** | **upper** | **overlap** | **mean** | **lower** | **upper** | **overlap** | **mean** | **lower** | **upper** | **overlap** | **mean** | **lower** | **upper** | **overlap** |
| **Northern (FI)** | **all** | **r** | 0.020 | 0.001 | 0.077 | 0.101 | 0.134 | 0.050 | 0.259 | 0.253 | 0.077 | 0.020 | 0.174 | 0.190 | 0.769 | 0.609 | 0.888 | 0.327 |
| **Northern (SWE)** | **all** | **r,i,p** | 0.069 | 0.009 | 0.179 | 0.202 | 0.163 | 0.057 | 0.295 | 0.289 | 0.035 | 0.001 | 0.121 | 0.144 | 0.733 | 0.601 | 0.845 | 0.294 |
| **Central (NGer)** | **spring** | **r** | 0.224 | 0.013 | 0.497 | 0.485 | 0.328 | 0.084 | 0.628 | 0.529 | 0.223 | 0.012 | 0.495 | 0.486 | 0.225 | 0.012 | 0.496 | 0.486 |
| **Central (NGer)** | **breeding** | **r,i,p** | 0.297 | 0.149 | 0.419 | 0.320 | 0.282 | 0.167 | 0.407 | 0.290 | 0.039 | 0.001 | 0.128 | 0.153 | 0.381 | 0.321 | 0.450 | 0.173 |
| **Central (NGer)** | **autumn** | **r** | 0.171 | 0.023 | 0.380 | 0.393 | 0.406 | 0.221 | 0.633 | 0.437 | 0.087 | 0.002 | 0.282 | 0.294 | 0.336 | 0.146 | 0.536 | 0.427 |
| **Southern (SGerCH)** | **spring** | **r** | 0.429 | 0.194 | 0.752 | 0.551 | 0.189 | 0.008 | 0.452 | 0.453 | 0.190 | 0.008 | 0.450 | 0.448 | 0.191 | 0.008 | 0.460 | 0.455 |
| **Southern (SGerCH)** | **breeding** | **r,i,p** | 0.454 | 0.281 | 0.639 | 0.393 | 0.414 | 0.245 | 0.581 | 0.378 | 0.061 | 0.002 | 0.204 | 0.220 | 0.070 | 0.025 | 0.138 | 0.151 |
| **Southern**  **(SGerCH)** | **autumn** | **r** | 0.448 | 0.246 | 0.705 | 0.485 | 0.312 | 0.134 | 0.496 | 0.408 | 0.079 | 0.009 | 0.226 | 0.240 | 0.161 | 0.040 | 0.346 | 0.347 |

Appendix Table 5: Estimated prevalences of Plasmodium lineages in the wintering areas – given the data collected in Africa south of the Sahara desert (Tab. 2). Indicated are the posterior mean, the 95% credible interval and the overlap between the prior and the posterior distribution for each Plasmodium lineage in every wintering area. Data from Bensch *et al.* 2000; Waldenström *et al.* 2002; Durrant *et al.* 2007; Beadell *et al.* 2009; Bonneaud *et al.* 2009; Chasar *et al.* 2009; Loiseau *et al.* 2010; Marzal *et al.* 2011; Loiseau *et al.* 2012; Hellgren *et al.* 2013; Mendes *et al.* 2013; Lutz *et al.* 2015; Sorensen *et al.* 2016.

| **Wintering area** | **Western Africa** | | | | **Central Africa** | | | | **Eastern Africa** | | | | **Southern Africa** | | | |
| --- | --- | --- | --- | --- | --- | --- | --- | --- | --- | --- | --- | --- | --- | --- | --- | --- |
| **Plasmodium lineage** | **mean** | **lower** | **upper** | **overlap** | **mean** | **lower** | **upper** | **overlap** | **mean** | **lower** | **upper** | **overlap** | **mean** | **lower** | **upper** | **overlap** |
| **SYBOR21** | 0.0063 | 0.0010 | 0.0137 | 0.0200 | 0.0004 | 0.0000 | 0.0016 | 0.0040 | 0.0023 | 0.0001 | 0.0088 | 0.0172 | 0.0054 | 0.0003 | 0.0132 | 0.0196 |
| **GRW02** | 0.0162 | 0.0085 | 0.0256 | 0.0289 | 0.0008 | 0.0001 | 0.0024 | 0.0049 | 0.0023 | 0.0001 | 0.0084 | 0.0161 | 0.0070 | 0.0016 | 0.0154 | 0.0227 |
| **GRW09** | 0.0033 | 0.0004 | 0.0090 | 0.0150 | 0.0288 | 0.0229 | 0.0352 | 0.0213 | 0.0550 | 0.0368 | 0.0765 | 0.0620 | 0.0018 | 0.0001 | 0.0063 | 0.0123 |
| **LINOLI1** | 0.0008 | 0.0000 | 0.0029 | 0.0065 | 0.0009 | 0.0002 | 0.0022 | 0.0043 | 0.0035 | 0.0004 | 0.0099 | 0.0171 | 0.0284 | 0.0192 | 0.0397 | 0.0338 |
| **PSEGRI1** | 0.0008 | 0.0000 | 0.0029 | 0.0067 | 0.0073 | 0.0046 | 0.0105 | 0.0111 | 0.0018 | 0.0001 | 0.0067 | 0.0139 | 0.0010 | 0.0000 | 0.0036 | 0.0077 |

Appendix Table 6: Estimated prevalences of Plasmodium lineages in the breeding populations – given the data collected in Europe and Africa (Tab. 2) and weighted by the estimated wintering area distribution of each focal population (Fig. 4, Tab. A4). Indicated are the posterior mean, the 95% credible interval and the overlap between the prior and the posterior distribution for each Plasmodium lineage in every wintering area. Data from von Rönn, Harrod, Bensch & Wolf 2015.

| **Breeding Population** | **Northern** | | | | **Central** | | | | **Southern** | | | |
| --- | --- | --- | --- | --- | --- | --- | --- | --- | --- | --- | --- | --- |
| **Plasmodium lineage** | **mean** | **lower** | **upper** | **overlap** | **mean** | **lower** | **upper** | **overlap** | **mean** | **lower** | **upper** | **overlap** |
| **SYBOR21** | 0.0131 | 0.0026 | 0.0288 | 0.0388 | 0.0123 | 0.0066 | 0.0199 | 0.0225 | 0.0104 | 0.0037 | 0.0212 | 0.0289 |
| **GRW02** | 0.0185 | 0.0068 | 0.0351 | 0.0436 | 0.0227 | 0.0140 | 0.0338 | 0.0322 | 0.0243 | 0.0127 | 0.0403 | 0.0443 |
| **GRW09** | 0.0237 | 0.0112 | 0.0411 | 0.0473 | 0.0347 | 0.0231 | 0.0489 | 0.0415 | 0.0494 | 0.0306 | 0.0741 | 0.0677 |
| **LINOLI1** | 0.0611 | 0.0402 | 0.0875 | 0.0710 | 0.0330 | 0.0225 | 0.0459 | 0.0376 | 0.0084 | 0.0038 | 0.0150 | 0.0198 |
| **PSEGRI1** | 0.0060 | 0.0023 | 0.0121 | 0.0185 | 0.0079 | 0.0043 | 0.0129 | 0.0159 | 0.0103 | 0.0055 | 0.0168 | 0.0198 |

References

Beadell, J.S., Covas, R., Gebhard, C., Ishtiaq, F., Melo, M., Schmidt, B.K., Perkins, S.L., Graves, G.R. & Fleischer, R.C. (2009) Host associations and evolutionary relationships of avian blood parasites from West Africa. *International Journal for Parasitology*, **39** (2), 257–266.

Bensch, S., Stjernman, M., Hasselquist, D., Ostman, O., Hansson, B., Westerdahl, H. & Pinheiro, R.T. (2000) Host specificity in avian blood parasites: a study of Plasmodium and Haemoproteus mitochondrial DNA amplified from birds. *Proceedings of the Royal Society B. Biological Sciences*, **267** (1452), 1583–1589.

Bonneaud, C., Sepil, I., Milá, B., Buermann, W., Pollinger, J., Sehgal, R.N.M., Valkiūnas, G., Iezhova, T.A., Saatchi, S. & Smith, T.B. (2009) The prevalence of avian Plasmodium is higher in undisturbed tropical forests of Cameroon. *Journal of Tropical Ecology*, **25** (04), 439–447.

Chasar, A., Loiseau, C., Valkiunas, G., Iezhova, T., Smith, T.B. & Sehgal, R.N.M. (2009) Prevalence and diversity patterns of avian blood parasites in degraded African rainforest habitats. *Molecular Ecology*, **18** (19), 4121–4133.

Durrant, K.L., Reed, J.L., Jones, P.J., Dallimer, M., Cheke, R.A., McWilliam, A.N. & Fleischer, R.C. (2007) Variation in haematozoan parasitism at local and landscape levels in the red-billed quelea Quelea quelea. *Journal of Avian Biology*, **0** (0), 071202183307004-0.

Hellgren, O., Wood, M.J., Waldenstrom, J., Hasselquist, D., Ottosson, U., Stervander, M. & Bensch, S. (2013) Circannual variation in blood parasitism in a sub-Saharan migrant passerine bird, the garden warbler. *Journal of Evolutionary Biology*, **26** (5), 1047–1059.

Loiseau, C., Harrigan, R.J., Robert, A., Bowie, R.C.K., Thomassen, H.A., Smith, T.B. & Sehgal, R.N.M. (2012) Host and habitat specialization of avian malaria in Africa. *Molecular Ecology*, **21** (2), 431–441.

Loiseau, C., Iezhova, T., Valkiunas, G., Chasar, A., Hutchinson, A., Buermann, W., Smith, T.B. & Sehgal, R.N.M. (2010) Spatial Variation of Haemosporidian Parasite infection in African Rainforest Bird Species. *The Journal of Parasitology*, **96** (1), 21–29.

Lutz, H.L., Hochachka, W.M., Engel, J.I., Bell, J.A., Tkach, V.V., Bates, J.M., Hackett, S.J. & Weckstein, J.D. (2015) Parasite prevalence corresponds to host life history in a diverse assemblage of afrotropical birds and haemosporidian parasites. *PLOS ONE*, **10** (4), e0121254.

Marzal, A., Ricklefs, R.E., Valkiunas, G., Albayrak, T., Arriero, E., Bonneaud, C., Czirjak, G.A., Ewen, J., Hellgren, O., Horakova, D., Iezhova, T.A., Jensen, H., Krizanauskiene, A., Lima, M.R., Lope, F. de, Magnussen, E., Martin, L.B., Moller, A.P., Palinauskas, V., Pap, P.L., Perez-Tris, J., Sehgal, R.N.M., Soler, M., Szollosi, E., Westerdahl, H., Zetindjiev, P. & Bensch, S. (2011) Diversity, loss, and gain of malaria parasites in a globally invasive bird. *PLOS ONE*, **6** (7), e21905.

Mendes, L., Pardal, S., Morais, J., Antunes, S., Ramos, J.A., Perez-Tris, J. & Piersma, T. (2013) Hidden haemosporidian infections in Ruffs (Philomachus pugnax) staging in Northwest Europe en route from Africa to Arctic Europe. *Parasitology Research*, **112** (5), 2037–2043.

Sorensen, M.C., Asghar, M., Bensch, S., Fairhurst, G.D., Jenni-Eiermann, S. & Spottiswoode, C.N. (2016) A rare study from the wintering grounds provides insight into the costs of malaria infection for migratory birds. *Journal of Avian Biology*, **47** (4), 575–582.

von Rönn, J.A.C., Harrod, C., Bensch, S. & Wolf, J.B.W. (2015) Transcontinental migratory connectivity predicts parasite prevalence in breeding populations of the European barn swallow. *Journal of Evolutionary Biology*, **28** (3), 535–546.

Waldenström, J., Bensch, S., Kiboi, S., Hasselquist, D. & Ottosson, U. (2002) Cross-species infection of blood parasites between resident and migratory sonbirds in Africa. *Molecular Ecology*, **11**, 1545–1554.
